# A Call for Urgent Action: Innovations for Nurse Retention in Addressing the Nursing Shortage

**DOI:** 10.3390/nursrep13010015

**Published:** 2023-01-25

**Authors:** Sonia Udod

**Affiliations:** College of Nursing, University of Manitoba, Winnipeg, MB R3T 2N2, Canada; sonia.udod@umanitoba.ca

The COVID-19 pandemic has become a defining moment for healthcare delivery and the health workforce despite “being prepared” for it. With each wave of the pandemic, the world and how we work has changed and we are becoming increasingly aware that we cannot fully return to what we knew in the workplace. More specifically, we find ourselves living in a liminal time, managing in-between realities in our journey from a familiar place to an unfamiliar destination. This editorial focuses on how we can leverage opportunities in this liminal context by innovating to address the global nursing shortage by focusing on nurse retention—assisting nurses working in hospitals to improve their work conditions so they can provide safe, quality patient care.

## 1. The Challenges

The pandemic intensified the existing problems of staffing shortages. Decades of neglect and a lack of funding have created unsafe working conditions and placed nurses in vulnerable situations, with limited options to practice physical and psychological self-care. Innovation in enhancing high-quality and safe working environments is needed so nurses can spend time directly providing the care that is desperately needed not only for patients but also for caregiver and families. As the largest proportion of the healthcare workforce, nurses apply their knowledge, skills, and experience to care for patients across the life span. Evidence shows that an increase in registered nurse staffing was linked with less hospital-related mortality, failure to rescue, and other adverse events (You et al., 2013 [1]).

A recent Statistics Canada survey (2022) [2] revealed that 9 in 10 nurses (92.0%) reported feeling more stressed at work than before the pandemic. Overtime hours among healthcare workers have been linked to decreases in physical and mental health and well-being, which can have long-term implications for the health of the workforce and impact the delivery of health services (Wong et al., 2019 [3]). A Canadian Federation of Nurses Union survey (2022) [4] found that 94% of nurses are suffering from symptoms of burnout. Feeling frustrated, overwhelmed, and lacking autonomy over their practice, many nurses have resigned their positions. Those nurses remaining in the profession have begun a “quiet quitting”, which is an often-misunderstood phrase that can mean either doing your job’s bare minimum or just not striving to overachieve (Detert, 2022 [5]). The pandemic has severely impacted nurses’ health and well-being and is impacting access to care.

## 2. Possible Approaches 

Charting the course of the future will depend upon redesigning and developing meaningful approaches to address the nursing shortage. Leary et al. (2022) [6] wrote an insightful paper on creating innovation infrastructure in schools of nursing to prepare nurses with the skills, knowledge, and experience necessary to lead in heath and healthcare innovation. Using Leary’s template, I offer the following approaches to enhance nurses’ work environments in practice. 

### 2.1. Establish Strategic Goals 

Establishing realistic and achievable goals (Leary et al., 2022 [6]) with nurses is an important first step in developing an innovation approach to improve nurses’ work environments. Innovation can mean different things to different disciplines but developing a disciplinary perspective of innovation by actively engaging frontline nurses is the first step in creating meaningful change. Nurses have a deep understanding of complex care processes, infection control practices, patient populations, and organizational structures of hospital units that can guide and inform innovative processes that leaders and others cannot (Dykes and Chu, 2020 [7]). This first step is key to laying the foundation to prioritize projects and activities, communicate initiatives with internal stakeholders, and serves as a benchmark against which progress is measured. The questions for consideration at this step can include: What skills do nurses need to maximize their impact in innovation and improve their work environments? What level and combination of nurses are needed (senior nurses, novice nurses)? Where and how will nurses be prepared for innovation? Most importantly, what other resources are required to support nurses in enhancing their work environments without additional strain on nurses? 

### 2.2. Build Infrastructure 

The goal in building the infrastructure to support innovation can ultimately improve care and services to enhance the patient’s and the nurse’s experience. Engaging nurses to enhance their practice requires support and cannot occur in isolation (McSherry and Douglas, 2011 [8]). First, resources must be put in place to develop advisory committees that include external and internal stakeholders, nurse leaders in innovative roles, nursing faculty, and, most importantly, nurses. Second, shaping the organizational culture to support innovation is dependent on a clear purpose, insightful people to generate ideas, and networking to extend internal and external partnerships in developing approaches to improving the work environment (Leary et al., 2022, [6]). The capacity of the organization to innovate is determined by multiple factors and generating and converting ideas into usable knowledge requires high levels of coordination and integration in partnership with organizational leaders. 

### 2.3. Focus on Inputs

We need to focus on inputs such as people, resources, and tools as opposed to outputs (i.e., staff engagement survey) where it is generally measured. Leary et al. (2022) [6] argue for maximizing leader development and identifying leader and nurse champions of innovation. This step lays a foundation for generating ideas but requires dedicated resources (time, money) to ensure changes are sustained. 

Establishing targeted workshops, seminars, and educational opportunities for nurses focused on innovation is one way to value and support nurses’ work environments. Inviting an innovator leader from a non-disciplinary perspective to spend time on the unit or within the organization may assist nurses to envision how innovation can be applied. For example, there may be additional ways to protect nurses time and energy from non-nursing duties that has taken valuable time away from direct patient care. 

Educational sessions can be used for peer-to-peer learning, community building, and moral support. Connecting nurses with other nurses allows them to share their expertise and frustrations. Moreover, in connecting with others through education and training, nurses can increase their capacity for psychological self-care and help protect themselves from moral distress.

### 2.4. Invest in Leadership Development

A culture of innovation requires strategic and forward-thinking leaders. Identifying learning opportunities and supporting leadership development is essential for current leaders and identifying qualified candidates to assume a leadership role as a part of succession planning. Creating a continuous learning environment can be demonstrated by adopting a special assignment to pursue one’s interests and develop skills, provide financial support for training, modify work schedules that free up time to attend workshops, and mentoring/coaching, as examples of leadership development. 

Positive role modelling and supportive leader behavior are necessary to build nurses’ trust. In a call to action by the Royal Society of Canada (2022) [9], rebuilding trust and valuing nurses are indispensable to safe quality patient care. Trust between leaders and nurses is necessary for meaningful innovations and can be facilitated through self-development techniques. 

## 3. Conclusions

To address our current nursing shortage, healthcare leaders and nurses must work together to enhance high-quality and safe working conditions through innovation to retain nurses. We must engage nurses; they know best. Will health leaders and policy makers embrace and engage the innovations that this pandemic necessitates? Collectively, we face a decision. We need to position nurses to drive and inform innovations in the healthcare system that advance their ability to provide safe quality care, not their ability to care for the organization. The appeal is urgent. 
